# Micelles of Progesterone for Topical Eye Administration: Interspecies and Intertissues Differences in Ex Vivo Ocular Permeability

**DOI:** 10.3390/pharmaceutics12080702

**Published:** 2020-07-26

**Authors:** Adrián M. Alambiaga-Caravaca, María Aracely Calatayud-Pascual, Vicent Rodilla, Angel Concheiro, Alicia López-Castellano, Carmen Alvarez-Lorenzo

**Affiliations:** 1 Departamento de Farmacia, Facultad de Ciencias de la Salud, Instituto de Ciencias Biomédicas, Universidad Cardenal Herrera-CEU, CEU Universities, C/Santiago Ramón y Cajal, s/n., Alfara del Patriarca, 46115 Valencia, Spain; adrian.alambiagacaravaca@uchceu.es (A.M.A.-C.); maria.calatayud@uchceu.es (M.A.C.-P.); vrodilla@uchceu.es (V.R.); 2 Departamento de Farmacología, Farmacia y Tecnología Farmacéutica, I+D Farma Group, Facultad de Farmacia, and Health Research Institute of Santiago de Compostela (IDIS), Universidade de Santiago de Compostela, 15782 Santiago de Compostela, Spain; angel.concheiro@usc.es

**Keywords:** progesterone, retinitis pigmentosa, interspecies ocular permeability differences, polymeric micelles, Soluplus, Pluronic, ocular drug delivery, solubility, hen’s egg-chorioallantoic membrane test (HET-CAM) assay

## Abstract

Progesterone (PG) may provide protection to the retina during retinitis pigmentosa, but its topical ocular supply is hampered by PG poor aqueous solubility and low ocular bioavailability. The development of efficient topical ocular forms must face up to two relevant challenges: Protective barriers of the eyes and lack of validated ex vivo tests to predict drug permeability. The aims of this study were: (i) To design micelles using Pluronic F68 and Soluplus copolymers to overcome PG solubility and permeability; and (ii) to compare drug diffusion through the cornea and sclera of three animal species (rabbit, porcine, and bovine) to investigate interspecies differences. Micelles of Pluronic F68 (3–4 nm) and Soluplus (52–59 nm) increased PG solubility by one and two orders of magnitude, respectively and exhibited nearly a 100% encapsulation efficiency. Soluplus systems showed in situ gelling capability in contrast to the low viscosity Pluronic F68 micelles. The formulations successfully passed the hen’s egg-chorioallantoic membrane test (HET-CAM) test. PG penetration through rabbit cornea and sclera was faster than through porcine or bovine cornea, although the differences were also formulation-dependent. Porcine tissues showed intermediate permeability between rabbit and bovine. Soluplus micelles allowed greater PG accumulation in cornea and sclera whereas Pluronic F68 promoted a faster penetration of lower PG doses.

## 1. Introduction

Retinitis pigmentosa (RP) is a degenerative disease that involves heterogeneous inherited photoreceptors apoptosis affecting the peripheral retina. RP is the most common hereditary retinal degeneration, causing blindness [1]. One of the concerns in RP is that a single mutation can cause a variety of different clinical phenotypes, so there is a large clinical and genetic heterogeneity; also, different mutations can cause the same syndrome [2]. In the early stages of the disease, rod photoreceptors are affected causing difficulties with dark adaptation, night blindness, and loss of mid-peripheral visual field in adolescence or young adulthood. As the RP advances and cones die, the central vision is lost. Nevertheless, RP is a highly variable disorder; some patients develop symptomatic visual loss in childhood whereas others remain asymptomatic until mid-adulthood [1,3,4].

Different therapeutic approaches have been adopted depending on the stage of the disease. In the early stages, the aim is to halt the degeneration using gene therapy or pharmacological treatments (neurotrophic growth factors or anti-apoptotic factors), reducing the production of retino-toxic molecules, and limiting oxidative damage. In advanced stages, when there are barely any functional photoreceptors left, retinal transplantation or electronic retinal implants may be needed [4]. In the early stages when rods die and oxygen consumption in the outer retina is reduced, the supply of oxygen is not decreased because there is no self-regulation of choroidal blood vessels. This leads to increased levels of oxygen in the external retina. It has been suggested that long-term protection against oxidative damage can delay the death of cones in patients with RP regardless of the causative mutation [5]. 

**Figure 1:**
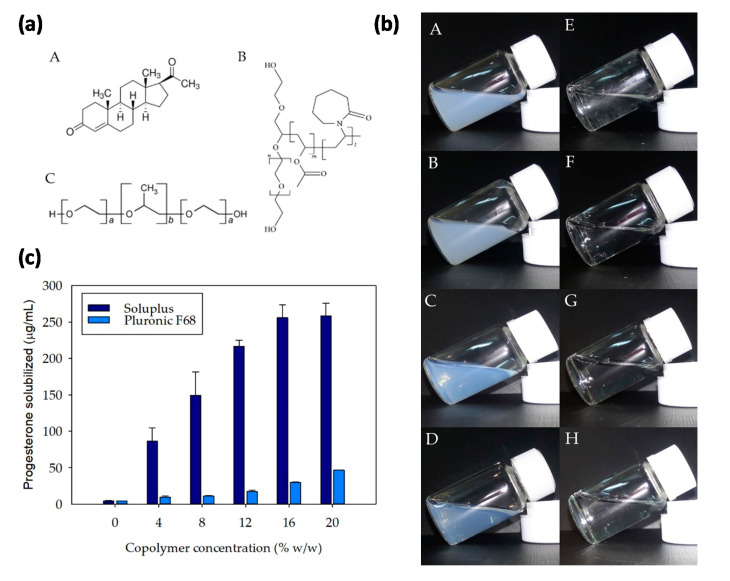
(**a**) Structure of progesterone (PG) (A), Soluplus^®^ (B), and Pluronic^®^ (C); (**b**) appearance of micelle dispersions (A) 16% Soluplus + PG, (B) 20% Soluplus + PG, (C) 16% Soluplus, (D) 20% Soluplus, (E) 16% Pluronic + PG, (F) 20% Pluronic + PG, (G) 16% Pluronic, and (H) 20% Pluronic; (**c**) PG solubility in Soluplus and Pluronic F68 micelle dispersions formulated in a phosphate saline buffer (PBS) pH 7.4. Error bars represent the standard deviations (*n* = 3).

Estrogens exert neuroprotection on the retina and optic nerve. Progesterone (PG; a C-21 steroid hormone, Figure 1a-A) and its derivatives may promote the formation of new myelin and reduce the extent of myelin sheath loss, showing therapeutic potential against retinal degeneration [6,7,8,9,10]. The retina is considered a true steroidogenic central nervous system (CNS) structure [9]. PG has been reported to promote cell survival and proliferation in non-neuronal tissues [10] and to provide protection to the retina during retinitis pigmentosa [11,12]. Recently, Hernandez-Rabaza et al. [13] studied the effects of PG on RP highlighting benefits such as reduction of the typical gliosis, decrease in the concentration of glutamate in the retina and increase of the concentration of glutathione. Similar results were found when PG administration was performed in the RP mouse model rd10. However, topical ocular delivery of PG is hindered by its poor aqueous solubility and low ocular bioavailability [13]. PG dosage forms for eye administration have barely been investigated and, to the best of our knowledge, there is only a report on PG encapsulation into nanocarriers [14]. That previous study revealed that the high affinity of PG for polybutylcianoacrylate nanoparticles causes that PG levels in ocular tissues were even lower than when applied as a solution, highlighting the need of an adequate design of the nanocarrier [14].

The development of safe and effective topical ocular formulations remains a challenging task due to the inherent anatomical and physiological barriers of the eye [15,16,17,18]. Topical administration is a painless, non-invasive, and simple practice method, but corneal tissue is a formidable barrier for drug penetration [5,6]. Corneal epithelium (lipophilic) limits the permeation of hydrophilic molecules, while the stroma, i.e., the layer below, is composed of a highly hydrated extracellular matrix that limits the permeation of lipophilic substances [1]. Moreover, instillation of eye drops activates defense mechanisms, such as lacrimation, which together with tear turnover, dilute the drug and clear it from the ocular surface. The excess volume is drained via the nasolacrimal duct into systemic circulation [3,5,6]. Overall, ocular drug bioavailability is less than 5% [19,20,21]. Polymeric micelles are an attractive option for ocular delivery due to their capability to encapsulate hydrophobic drugs, facilitating the development of clear aqueous solutions that avoid the sticky sensation and blurred vision of semisolid formulations. The amphiphilic excipients used in micelles can facilitate the permanence of the formulation on the eye surface and inhibit efflux pumps on corneal tissue [22]. Moreover, micelles can facilitate drug penetration through both the cornea and sclera, providing therapeutic drug levels to the back of the eye [23,24].

The preclinical assessment of ocular drug delivery systems commonly involves ex vivo permeability studies using non-human cornea or sclera. The availability of each animal species is different according to the region [25] and thus ex vivo tests of human formulations have been reported in the literature for a variety of animal tissues, mainly rabbit, porcine, and bovine [26]. These tests in animal tissues are mainly intended to predict human drug permeability assuming that the test renders similar results disregarding the species. Rabbit cornea and sclera have been preferred in the majority of ex vivo permeation studies in order to correlate with in vivo preclinical tests of safety and efficacy carried out also in rabbits, although now these in vivo tests are being restricted by most Animal Ethical Committees over the world [4]. Porcine eye tissues seem to be the most similar to the human ones: The eyeballs have similar weight and size, and also the volumes of aqueous humor and the vitreous body are comparable [27]. The permeability of certain active substances through the porcine cornea is estimated to be similar or slightly lower than through the human cornea since the porcine corneal epithelial layer is a little thicker. In parallel, the permeability of bovine corneas may be lower because bovine epithelium has almost twice as many cell layers as the human corneal epithelium [27]. Despite differences between human and bovine corneas, the Bovine Cornea Opacity/Permeability test (BCOP) is one of the currently approved alternatives to in vivo ocular irritation assay in rabbits [28]. There is only one study that reports on the interspecies (porcine, rabbit, and bovine) differences in the apparent permeation coefficient, *P*_app_, of ciprofloxacin hydrochloride, lidocaine hydrochloride, timolol maleate, and dexamethasone dissolved in a buffer solution. *P*_app_ values were recorded for each drug through sclera, cornea, conjunctiva, choroid and retina, and the combination of the different tissue layers. As expected, disregarding the drug and the animal, *P*_app_ was higher for conjunctiva and sclera and lower for cornea. The values recorded for rabbits were in general higher and also showed greater variability than those recorded for porcine tissues. Moreover, tissue dissimilarities were proved not to be the only cause of the *P*_app_ differences. Drug lipophilia (log P between 1 and 3) seems to facilitate the penetration in all tissues investigated [21,26]. The cornea has a molecular weight cut-off of 400–600 Da while sclera allows the pass of 70 kDa molecules [29].

The aim of the present study was two-fold: (i) To design micelle-based formulations of PG that can overcome the drug solubility and permeability limitations when intended for topical treatment of RP; and (ii) to compare drug diffusion through cornea and sclera of three animal species, namely, rabbit, porcine, and bovine, in order to investigate interspecies differences. Specifically, two polymers, Soluplus and Pluronic F68, were selected to prepare the micelles. Soluplus is a polyvinyl caprolactam—polyvinyl acetate—polyethylene glycol graft copolymer (PCL-PVAc-PEG) (Figure 1a-B). Soluplus has a very low critical micelle concentration (CMC) value (6.6 × 10^−5^ mM) and its micelles are highly stable against dilution and may also undergo in situ gelling on the ocular surface, enhancing penetration into ocular structures [23,30]. The hydrophobic core of Soluplus micelles allows the encapsulation of poorly soluble drugs, notably enhancing their apparent solubility [31]. Pluronic F68 is a linear triblock poly(ethylene oxide)—poly(propylene oxide)—poly(ethylene oxide) (PEO-PPO-PEO) copolymer (Figure 1a-C) that, in addition to the self-assembling properties, exhibits the capability to inhibit P-glycoprotein efflux pumps at the eye surface and to undergo sol-gel transitions upon heating [30]. To carry out the study, first the capability of micelles prepared covering a wide range of copolymers concentrations to solubilize PG was investigated. After a screening of ocular compatibility using the HET-CAM test, ex vivo drug permeability through the cornea and sclera from the three selected animal species was investigated.

## 2. Materials and Methods

### 2.1. Materials

Progesterone (PG, 314.46 g/mol), Pluronic^®^ F68 (8350 g/mol), and PG: Methyl-β-cyclodextrin complex (85.2 mg PG/g; Progresterone-water soluble) were purchased from Sigma-Aldrich Chemical Co. (St. Louis, MO, USA), Soluplus^®^ (115,000 g/mol) from BASF (Ludwigshafen, Germany), acetonitrile was provided from Scharlab SL (Barcelona, Spain), ethanol absolute from VWR (Fontenay-sous-Bois, France), propylene glycol from Guinama (La Pobla de Vallbona, Spain). Ultrapure water was obtained by reverse osmosis (Milli-Q, Millipore Ibérica, Madrid, Spain). All other chemicals used were of analytical reagent grade and used as received.

### 2.2. Micelles Preparation and Characterization

Micelles were prepared by dispersing Soluplus or Pluronic F68 at different concentrations (4%, 8%, 12%, 16%, and 20% *w*/*w*) in triplicate in a phosphate saline buffer (PBS) pH 7.4. The dispersions were kept under magnetic stirring for 24 h at room temperature. The size, zeta potential, and polydispersion index (PDI) of the micelles (filtered through 0.22 µm membranes; Acrodisc^®^ Syringe Filter, GHP Minispike, Waters, Milford, MA, USA) were measured in triplicate in a Zetasizer^®^ 3000HS (Malvern Instruments, Worcestershire, UK). The pH was measured using a calibrated GLP22 pH meter (Crison Instruments, L’Hospitalet de Llobregat, Spain) [24].

### 2.3. Solubility of Progesterone (PG) in Micelle Dispersions

Aliquots (10 mL) of Soluplus and Pluronic F68 dispersions prepared as above were placed in tubes containing PG (2.6–3.0 mg) and kept under constant magnetic agitation (Unitronic, JP Selecta, Barcelona, Spain) at 300 rpm and room temperature [24,30]. The PBS pH 7.4 medium without a copolymer was used as the control. After 96 h of stirring, the dispersions were centrifuged (centrifuge model 5804R, Eppendorf AG, Hamburg, Germany) at 5000 rpm for 30 min to separate the non-solubilized PG. The supernatants were collected gently and diluted with an ethanol:water (30:70 *v*/*v*) mixture to determine the apparent solubility of PG. The experiments were carried out in triplicate.

The PG concentration was measured in a HPLC (AS-4140 autosampler, PU-4180 pump, LC-NetII/ADC interface box, CO-4060 column oven, MD-4010 photodiode array detector) from Jasco (Tokyo, Japan) fitted with a C18 column (LiquidPurple ODS C18, 5 µm, 4.6 × 150 mm) and operated using the ChromNAV software (ver. 2, Jasco, Tokyo, Japan). The mobile phase was acetonitrile:water (20:80 *v*/*v*) at 1 mL/min and 25 °C. The injection volume was 50 µL. PG was quantified at 245 nm (retention time 5.1 min). Standard solutions of PG (0.01–5 µg/mL) in ethanol/water (30:70 *v*/*v*) were prepared.

The solubilization capability was characterized taking into account PG solubility in the medium without (*S_W_*) and with micelles (*S_tot_*), the concentration of the copolymer used (*C_copol_*), the CMC of the copolymer, and the universal constant of gases (*R*), as follows [24,30]. The molar solubilization capacity (moles of drug solubilized per mol of copolymer forming micelles):

(1) $$X=\frac{S_{tot}-S_{w}}{C_{copol-CMC}}$$

The micelle-water partition coefficient (ratio between the drug concentration in the micelle and the aqueous phase):

(2) $$P=\frac{S_{tot}-S_{w}}{S_{w}}$$

The molar micelle-water partition coefficient, assigning a default copolymer concentration of 1 M:

(3) $$PM=\frac{X\left(1-CMC\right)}{S_{w}}$$

Gibbs standard-free energy of solubilization was estimated from the molar micelle/water partition coefficient (*PM*) and the micelle-water partition coefficient (*P*), as:

(4) ΔGs=−RT×ln(PM)

(5) ΔGs=−RT×ln(P)

The proportion of drug molecules encapsulated in the micelles:

(6) $$mf=\frac{S_{tot}-S_{w}}{S_{tot}}$$

### 2.4. Rheological Analysis

The dependence on temperature of storage (G′) and loss (G′′) moduli of Soluplus and Pluronic F68 dispersions was evaluated in a Rheolyst AR-1000N rheometer (TA Instruments, Newcastle, UK) equipped with an AR-1000N data analyzer, a Peltier plate, and cone geometry (59 mm diameter, 2.1°). The angular frequency was fixed at 5 rad/s and the temperature increased from 15 to 40 °C with a ramp rate of 2 °C/min, while the oscillation stress was kept at 0.1 Pa.

### 2.5. Hen’s Egg Test Chorioallantoic Membrane (HET-CAM)

Fertilized hen’s eggs from the Coren Technological Incubation Center (San Cibrao das Viñas, Spain) were incubated at 37 °C and 60% RH (Ineltec, model CC SR 0150, Barcelona, Spain). Each of the eggs was manually rotated 180° every 8 h to ensure the correct embryo development. After nine days of incubation, a circular cut (1 cm) was made in the air chamber of the eggshell. The inner membrane was moistened with 0.9% NaCl for 30 min and then carefully removed to expose the chorioallantoic membrane (CAM). Dispersions of Soluplus and Pluronic F68 micelles loaded with PG (200 μL) were deposited in the CAM of different eggs. Solutions of 0.1 N NaOH and 0.9% NaCl were used as positive and negative controls, respectively (300 μL). The eggs were monitored for bleeding, vascular lysis, and coagulation of the CAM vessels for 300 s. The irritation score (IS) was calculated as:

(7) $$IS=\frac{\left(301-t_{H}\right)\times 5}{300}+\frac{\left(301-t_{L}\right)\times 7}{300}+\frac{\left(301-t_{C}\right)\times 9}{300}$$

In this equation, *t_H_* represents haemorrhage time (s), *t_L_* is lysis time (s) and *t_C_* is coagulation time (s) [30]. According to the IS values, the substances are classified as non-irritant (*IS* < 1), mildly irritant (1 ≤ *IS* < 5), moderately irritating (5 ≤ *IS* < 10), or severely irritant (*IS* > 10) [32].

### 2.6. Ex Vivo Corneal and Sclera Permeability Assay

Bovine, porcine, and rabbit eyes were supplied by a local slaughterhouse and transported immersed in a PBS medium without antibiotics in an ice bath. Corneas and scleras were carefully isolated, washed with PBS, and used to separate donor and receptor compartments in vertical diffusion Franz cells [33,34,35,36,37].

The receptor chamber was filled with propyleneglycol:water (40:60% *w*/*w*) pH 7.4 (6 mL). The donor chamber (2 mL) was filled with the same solution and the tissue was allowed to balance for 1 h. The solubility of PG in the receptor medium has already been established [38] and ensured sink conditions. The receptors were kept at 35 °C (temperature controlled bath), and gentle magnetic stirring was applied for 1 h. After 1 h, the solution at the donor chamber was completely removed using a Pasteur pipette and replaced by the micelles formulations (1 mL) prepared as described above in a PBS pH 7.4. The donor compartments (0.785 cm^2^ permeation available area) were covered with parafilm. Samples (1 mL) were taken manually from the receptor chamber at 30, 60, 90, 120, 180, and 240 min, and replaced with a fresh medium avoiding bubbles. All the experiments were carried out in quadruplicate. As a control, a solution of PG (343.04 μg/mL) was prepared by dispersion of the commercially available PG: Methyl-β-cyclodextrin complex powder (85.2 mg PG/g) (Sigma-Aldrich Co.; St. Louis, MO, USA) in a buffer pH 7.4 and evaluated in parallel.

PG permeated was analyzed by HPLC and the cumulative amount of the drug (μg/cm^2^) collected in the receptor medium was plotted against time to estimate the apparent permeability coefficients (*P*_app_, cm/s), as follows:

(8) $$P_{\mathrm{app}}=\frac{\Delta Q}{\Delta t}\times {\left(A\times Co\times 60\right)}^{-1}$$

In this equation, Δ*Q*/Δ*t* (μg/min) is the steady state flow (J) across corneal or scleral tissue, *A* is the exposed surface area of ocular tissue (0.785 cm^2^ in bovine and porcine tissues; 0.567 cm^2^ in rabbit), *C*_0_ is the initial PG concentration (μg/mL) in the donor compartment, and 60 is taken as the factor to convert minute into second [30,39].

Each experiment was performed in triplicate, and the results were reported as the mean value ± standard deviation. The steady state flow (J) was calculated from the slope as well as the lag time (*T*_0_) by intersecting with the *x*-axis of the linear regression [31]. The drug concentration in the donor chamber was also quantified at the end of the test (4 h). All corneas and scleras were visually inspected after the test to verify that all of them were in good conditions, without cracks or changes in their appearance. Corneas and scleras were placed in Falcon tubes containing acetonitrile (2 mL). The tubes were kept under mild stirring at 37 °C for 24 h, immersed in an ultrasound bath for 99 min at 37 °C, and centrifuged (1000 rpm, 5 min, 25 °C). The supernatants were filtered (Acrodisc^®^ Syringe Filter, 0.22 µm GHP Minispike, Waters), centrifuged (14,000 rpm, 20 min, 25 °C), and filtered to be analyzed in HPLC.

### 2.7. Statistical Analysis

Data analysis was performed using the Mann-Whitney or the Kruskal-Wallis tests as appropriate. Post-hoc multiple comparisons were carried out using the Man-Whitney test applying Bonferroni’s correction for significance. Statistical analysis was carried out using SPSS 24.0.

## 3. Results and Discussion

### 3.1. Micelles Preparation and PG Solubilization

Both Pluronic and Soluplus copolymers (Figure 1a-B,a-C) are attractive excipients for ocular drug delivery owing to their self-assembling properties, which enable forming micelles and also in situ gelling systems at moderate concentrations. Compared to Pluronic, the literature on the applications of Soluplus to formulate eye drops is more limited. Soluplus has the advantage of its extremely low CMC (6.6 × 10^−5^ mM vs. 4.0 × 10^−2^ mM for Pluronic F68) [40] because of the higher hydrophobicity of the caprolactam moieties, and thus Soluplus micelles are more stable against dilution [41]. Moreover, differently, Soluplus solutions in water are slightly acid (pH 5.6) while Pluronic F68 solutions are neutral (pH 7.4). Although these pH values are well tolerated by the eye surface, for comparison purposes all micelle dispersions were prepared in a buffered medium. Accordingly, Soluplus and Pluronic F68 micelles were prepared in a PBS pH 7.4 covering a wide range of copolymer concentrations (4, 8, 12, 16, and 20% *w*/*w*) (Table 1). The concentrations of each copolymer chosen for the study were well above the CMC values, and ranged between 0.35 and 1.74 mM for Soluplus, and 4.79–23.95 mM for Pluronic F68 (corresponding to 4–20% *w*/*w* concentration for both copolymers).

Unimodal number-based size distributions were observed for all micelle dispersions (Figure S1). Soluplus micelles had an average size of 52.32 ± 10.13 nm and a polydispersity index of 0.24 ± 0.01, while Pluronic F68 micelles were much smaller showing an average size of 3.74 ± 1.13 and a polydispersity index of 0.53 ± 0.08 (Table S1 in Supplementary Material). The PG load did not cause a relevant change in the size of the micelles (Table 1). The surface charge of all polymeric micelles was slightly negative or near zero according to the composition of the two copolymers.

The initial transparent (Pluronic F68) or opalescent (Soluplus) dispersions kept the same appearance when adding PG (Figure 1b). Pluronic produced clearer formulations due to its high hydrophilic-lipophilic balance (HLB = 29), while Soluplus dispersions opalescence is related to that the aggregates are larger and less hydrophilic (HLB = 16). The transmittance of 20% Soluplus dispersion in a PBS pH 7.4 measured using a 10 mm light path cuvette is shown in Figure S2 (Supplementary Materials). Dispersions including PG behaved similarly. The transmittance of a liquid layer of Soluplus/PG system of about 10 microns, which corresponds to the thickness of the human tear film [42], can be estimated to be close to 100% in the visible light range. Therefore, no change in visual acuity is expected after instillation.

Once the micelles were prepared, their capability to encapsulate PG was investigated. PG solubility in a PBS at 25 °C was 4.4 μg/mL, which was lower than the solubility in water reported in the literature, i.e., 7.0 μg/mL [43]. In the presence of the copolymers, PG solubility experienced a remarkable increase (Figure 1c). Pluronic F68 micelles (20% in PBS) increased the apparent solubility up to 46.8 ± 0.2 µg/mL. In the case of Soluplus (20% in PBS) the increase was even more remarkable; an apparent solubility of 258.2 ± 17.6 µg/mL was recorded and the remaining solid PG was not detected. This means that Soluplus 20% micelles dispersions could even encapsulate larger amounts of PG. Parameters used to quantify the solubilization efficiency are summarized in Table 2 and Table 3. Micelle-water partition coefficients, expressed as log P-values, ranged between 3 and 4 for Pluronic micelles and between 4 and 5 for Soluplus micelles. These values clearly indicated that the proportion of PG encapsulated inside the micelles is to 3-to-5 orders of magnitude higher than the free drug remaining in the aqueous medium. Indeed, an increase in the copolymer concentration favored the encapsulation of more PG molecules, and according to the mf values (Table 2), more than 99.9% of drug was hosted inside the micelles for the higher Soluplus concentrations tested. Therefore, the outstanding encapsulation efficiency of ca. 100% means that almost no free drug is outside the micelles (when solubilization equilibrium was reached and excess of solid drug was removed). The huge range of PG amounts that can be solubilized using Pluronic and Soluplus micelles may offer an enormous versatility to formulate the dose required for each patient.

The free energy of solubilization was negative in all cases; probably the encapsulation process was thermodynamically driven by hydrophobic interactions with the micelle cores. So far the largest increases in PG solubility have been reported for cyclodextrins in a PEG:water medium, attaining values close to 1 mg/mL [43]. The remarkable advantages of PG encapsulation in the prepared polymeric micelles compared to the inclusion complex formation with cyclodextrins rely on: (i) The outstanding 100% encapsulation efficiency in micelles, which is never reached with cyclodextrins; (ii) the higher thermodynamic and kinetic stability of the micelles, which are less prone to release the encapsulated drug than the cyclodextrins [44,45], and (iii) that organic cosolvents are not needed.

### 3.2. Rheological Properties of the Formulations

The rheological properties of a topical ocular formulation notably determine the time of permanence on the ocular surface and, in turn, the drug ocular bioavailability [46]. The use of thickening agents is frequent, but their concentration requires a fine balance between the required flowability of the liquid formulation during dosing (viscosity under shearing conditions below 25 × 10^−3^ Pa·s) and the consistency demanded to remain on the eye (shear viscosity ≥ 10 × 10^−3^ Pa·s) [47]. The upper limit of viscosity could be expanded by means of polymers that undergo in situ gelling phenomena [48]. Thus, the next step was to investigate whether the presence of the micelles can increase by themselves the viscosity of the formulation and to determine the effect of temperature on the rheological properties.

**Figure 2:**
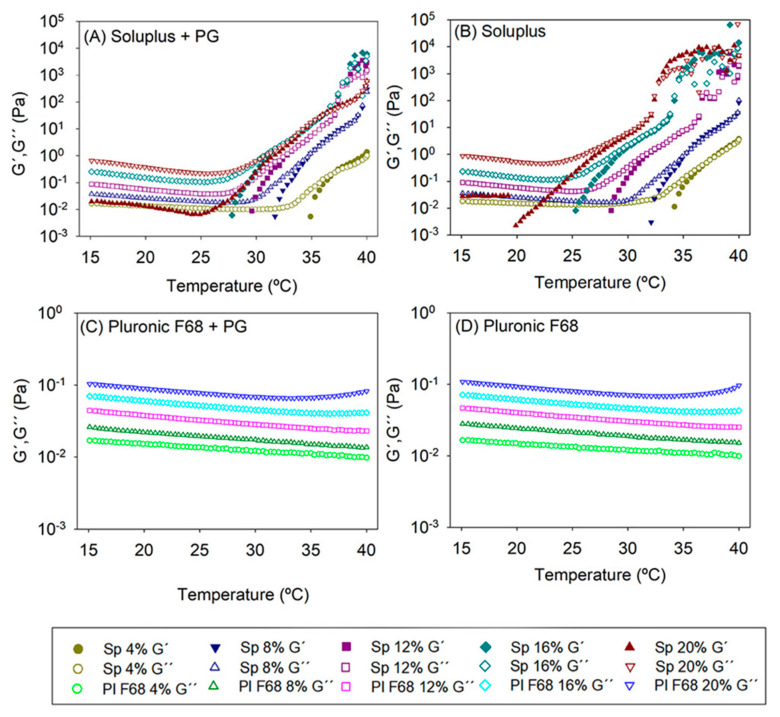
Evolution of the storage (G′) and the loss (G′′) moduli as a function of temperature of (**A**) PG-loaded Soluplus micelle dispersions, (**B**) blank Soluplus dispersions, (**C**) PG-loaded Pluronic F68 micelle dispersion, (**D**) blank Pluronic dispersions. Total copolymer concentration was 4, 8, 12, 16, and 20% *w*/*w* in a PBS pH 7.4 buffer (*n* = 4).

Viscoelastic behavior of copolymer dispersions with and without PG is depicted in Figure 2. Pluronic F68 dispersions (4–20% *w*/*w*) with and without PG showed a viscous behavior with negligible storage modulus (G′) in the 15–40 °C range. The G′′ values were constant in the temperature interval evaluated and no sol-gel transition was observed. The presence of PG caused minor changes in the loss modulus of Pluronic F68. Differently, Soluplus blank dispersions exhibited the typical sol-gel transition showing a remarkable increase in G’ when a certain temperature was reached (Figure S3 in Supplementary Materials). The transition temperature decreased as the copolymer concentration increased: 4%, 37.5 °C; 8%, 35.6 °C; 12%, 35.7 °C; 16%, 34.6 °C, and 20%, 32.7 °C. For the PG-loaded micelle dispersions the transition temperature was: 4%, 37.4 °C; 8%, 34.9 °C; 12%, 33.5 °C; 16%, 36.3 °C, and 20%, 35.6 °C. Nearby and above the transition temperature both G′ and G′′ showed a progressive increase in their values. This temperature-dependence pattern agreed well with other reports on Soluplus dispersions [26] and was quite different to the typical in situ gelling behavior reported for other temperature-responsive copolymers that exhibit brusque transitions. Interestingly, at 35 °C Soluplus 12–20% micelle dispersions could transform into soft gels on the eye surface prolonging the retention time and the drug release. The complex viscosity values (i.e., the frequency-dependent viscosity) recorded at 35 °C for Soluplus 16% and 20% in a PBS with PG were 4.08 and 4.63 Pa·s, respectively, while for Pluronic 16% and 20% in a PBS with PG, the values of complex viscosities were 9.87 × 10^−3^ and 14.21 × 10^−3^ Pa·s, respectively. These findings indicate that Pluronic micelles communicate shear viscosity above the critical level to maintain precorneal residence in man [47].

### 3.3. HET-CAM Test

A first screening of ocular compatibility was carried out placing the formulation in contact with the hen´s egg chorioallantoic membrane (HET-CAM). This test is an alternative to the in vivo Draize test to evaluate the irritancy that ocular formulations may cause [30,31,49]. CAM is a fetal membrane that is not innervated but highly vascularized and responds to injury similarly to the rabbit conjunctiva made of fused chorion and allantois [50]. The effects induced by the substance to be tested on the small blood vessels and proteins of this soft tissue membrane are used as an indicator of the effects induced by the same test substance on the eye of a treated rabbit [32].

**Figure 3:**
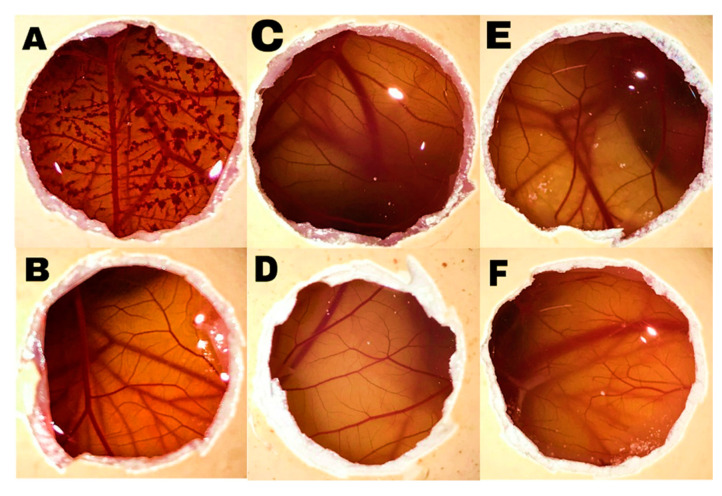
Effect of controls and test substances on the surface of the chorioallantoic membrane (CAM) after treatment for 3 min (**A**) NaOH 0.1 N, (**B**) NaCl 0.9% *w*/*w*, (**C**) Soluplus (16%), (**D**) Soluplus (20%), (**E**) Pluronic F68 (16%), and (**F**) Pluronic F68 (20%) dispersions in a PBS.

The HET-CAM test revealed that Soluplus and Pluronic F68 micelle dispersions behaved as the negative control (0.9% NaCl) without inducing bleeding, lysis or coagulation at the time of the study (*t*_H_, *t*_L_, and *t*_C_ > 301 s; *IS* = 0), which means that they can be considered non-irritants (Figure 3). The IS for the positive control (0.1 N NaOH) was 18.73 (*t*_H_ =30 s, *t*_L_ = 30 s, *t*_C_ = 35 s). The non-irritant behavior of the Soluplus and Pluronic F68 micelle dispersions is in good agreement with previous reports on HET-CAM assay results recorded for 23% *w*/*v* Soluplus dispersions [31] and in situ gel formulations prepared with 23% *w*/*v* Pluronic F127 and 15% *w*/*v* Pluronic F68 [51].

### 3.4. Ex Vivo Permeation Assay

First, pig eyes were selected as they are structurally similar to human eyes in terms of globe size, corneal thickness, ratio of globe diameter to corneal length, presence of Bowman’s layer and sclera histology, and collagen bundle organization [25,52]. PG permeability studies were first carried out for both 16% and 20% polymer concentrations with the aim of identifying the formulation that promoted the highest drug penetration through cornea and sclera. Formulations had a PG load of 256 and 258 μg/mL for Soluplus 16% and 20% dispersions, respectively. In parallel, Pluronic F68 16% and 20% dispersions contained 30.1 and 46.8 μg/mL, respectively.

**Figure 4:**
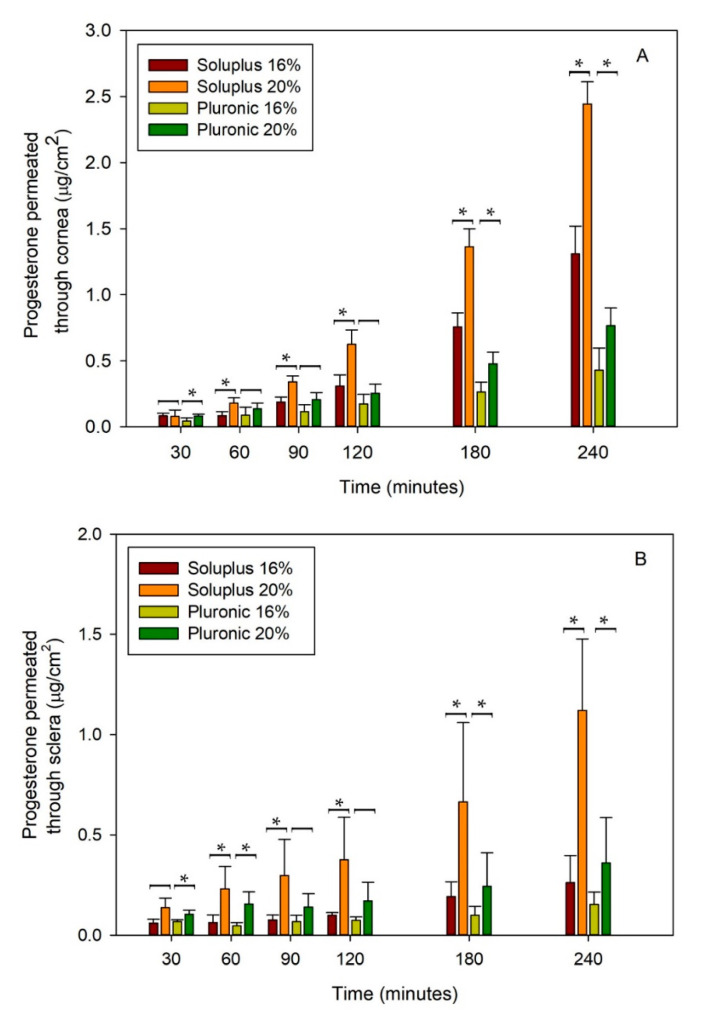
Trans-corneal (**A**) and trans-scleral (**B**) permeation of PG as a function of time when formulated in Soluplus or Pluronic F68 micelles (*n* = 4). * Significant differences were found between 16% and 20% of either Soluplus or Pluronic.

After 4 h in contact with cornea, the amounts of PG accumulated in the receptor chamber were 1.31 ± 0.21 and 2.44 ± 0.17 µg/cm^2^ when applied as Soluplus micelles at 16% and 20%, respectively. In the case of Pluronic 16% and 20% micelle dispersions, the amounts accumulated were 0.43 ± 0.17 and 0.77 ± 0.13 µg/cm^2^, respectively (Figure 4A).

In parallel, the amount of PG that passed through the sclera in 4 h was 0.26 ± 0.14 and 1.12 ± 0.36 µg/cm^2^ for Soluplus micelles at 16% and 20%, respectively, and 0.15 ± 0.06 and 0.36 ± 0.23 µg/cm^2^ for Pluronic micelles at 16% and 20%, respectively (Figure 4B). Thus, differently to the common trend observed for most ophthalmic drugs in which sclera permeability is higher than cornea permeability [53], in the present study PG permeability through cornea was somewhat favored. It has been previously observed that sclera and cornea permeabilities become similar when the drug is hydrophobic and, therefore, exhibits high affinity for the hydrophobic epithelium of cornea and low affinity for the aqueous porous network of sclera [28]. Indeed, it should be noticed that PG is a BCS Class 2 drug, i.e., it exhibits low solubility (nearly insoluble in a buffered medium) and high permeability. PG is a small molecule (MW 314.46 g/mol) with a very low polar surface area (log P = 3.58) [54]. Interestingly, although the BCS classification refers to intestinal permeability, hydrophobic drugs appear as the most suitable to cross most biological membranes and therefore the BCS class may provide preliminary information about the feasibility of a drug that could pass through the cornea [28]. In the particular case of a hydrophobic drug, such as PG, encapsulated in micelles, additional factors should be considered. Micelles in contact with the cornea provide an enhancement in drug gradient concentration and may facilitate the partition of the drug towards the epithelium, but also the drug-loaded micelles may easily cross the stroma [55]. In the case of sclera and for a drug so hydrophobic as PG, only the drug encapsulated inside the micelles is expected to pass through the tissue, in which the arrangement of the collagen bundles allows the formation of a pore network (20–200 nm) full of water that screens the crossing of the substances as a function of their molecular size [56]. As expected, formulations that provided higher PG apparent solubility led to higher amounts of drug permeated.

The steady state flux (J) of PG was estimated to be 0.50 ± 0.07, 0.91 ± 0.04, 0.13 ± 0.07, and 0.26 ± 0.04 μg/(cm^2^·h) for Soluplus 16% and 20% and Pluronic 16% and 20%, respectively through cornea; and 0.08 ± 0.06, 0.37 ± 0.08, 0.04 ± 0.03, and 0.09 ± 0.07 μg/(cm^2^·h) for Soluplus 16% and 20% and Pluronic 16% and 20%, respectively through sclera. Statistical differences (*p* < 0.05) were detected at various times between different concentrations of each polymer used as illustrated in Figure 4A,B. As permeability values recorded through cornea and sclera were larger for Soluplus and Pluronic 20% micelle dispersions, these formulations were selected for the studies in different species.

Bovine eyes are significantly larger than human eyes; but they are widely used in diffusion studies too [24,31,53,57,58]. Bovine corneal epithelium has been reported to be at least two times thicker than the human one, which may lead to lower drug permeability [27]. Rabbit eyes have been widely used for ex vivo models [33,59,60,61], although they are smaller than human eyes and lack from Bowman’s layer, which may facilitate drug penetration [27]. To elucidate the potential impact of these differences on PG permeability results, the thicknesses of the cornea and sclera tissues of the as-supplied eyes were measured using a caliper. Cornea thickness was 91.2 ± 0.8, 87.0 ± 2.1, and 51.7 ± 7.1 μm for bovine, porcine, and rabbit, respectively. Sclera thickness was 129.8 ± 14.7, 73.2 ± 2.7, and 24.3 ± 4.9 μm for bovine, porcine, and rabbit, respectively.

**Figure 5:**
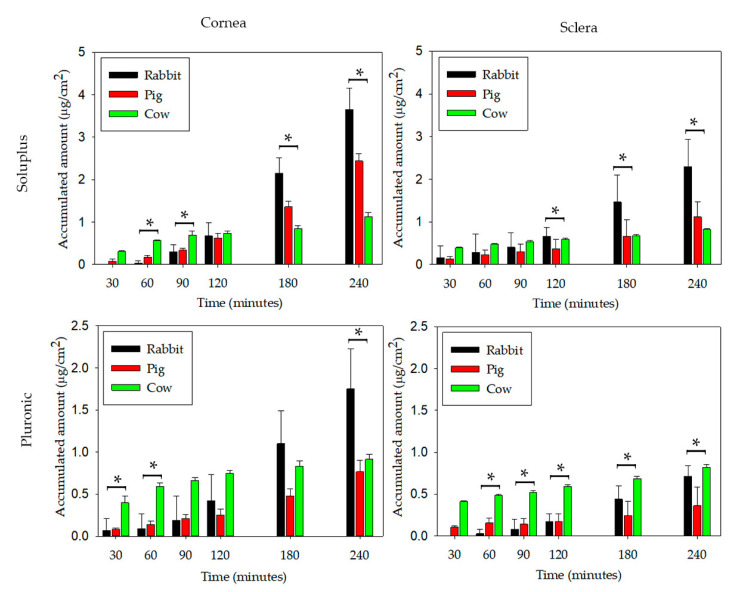
Accumulated amount of PG permeated through rabbit, porcine and bovine cornea, and sclera and measured in the receptor chamber as a function of time. PG was formulated in Soluplus and Pluronic micelles, total copolymer concentration was 20% *w*/*w* in all cases (*n* = 4). * Significant differences were found between the three species (*p* < 0.05).

In the case of PG-loaded Soluplus micelles, the amounts accumulated in the receptor compartment of cornea and sclera ranked as follows: Rabbit > porcine > bovine (Table 4). Differently, Pluronic micelles did not follow such a clear trend; the amount of drug that passed through cornea ranked rabbit > porcine ~ bovine, while in the case of sclera ranked rabbit ~ bovine > porcine (Figure 5). In the three species, the amount of PG permeated was higher through cornea than through sclera (*p* < 0.05).

Statistical differences were also detected between the different species under investigation and in both corneas and scleras (Figure 5). The amounts accumulated were higher for Soluplus than Pluronic micelle dispersions in good agreement with the higher amount of PG supplied.

**Figure 6:**
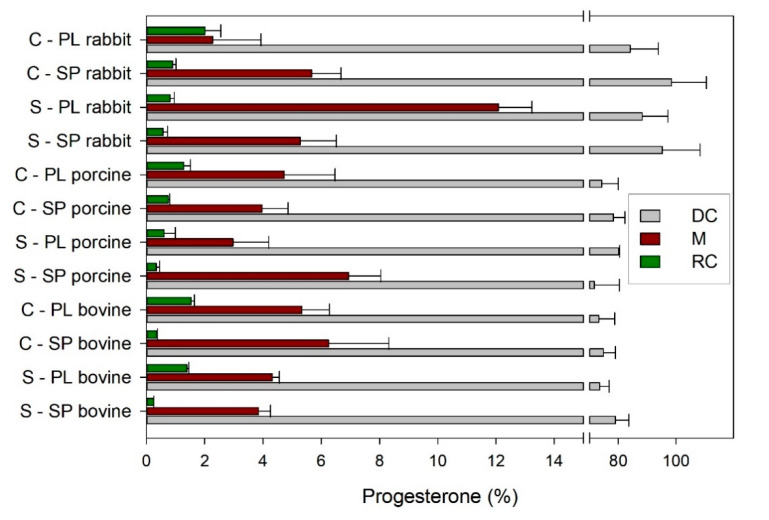
Mass balance, percentage (%) of PG accumulated in the donor compartment (DC); in the membrane (M) of bovine, porcine, and rabbit and in the receiving compartment (RC) after 4 h of experiment of permeation of micelles Soluplus (SP) and Pluronic (PL) in corneas (C) and scleras (S). The total polymer concentration was 20% *w*/*w* in all cases (*n* = 4).

After the 4 h assay, corneas and scleras were visually inspected and all were in good condition, without holes or cracks. The mass balance was determined from the concentrations in the donor and receptor compartment and in the membrane after the 24 h extraction (Figure 6).

The coefficient of apparent permeability (*P*_app_) of PG through the cornea and sclera of the three animal species was calculated from the flow ratio (J) and the concentration of PG in the donor phase (Table 5). The smallest permeability coefficient was for Soluplus micelles in sclera bovine, while the highest *P*_app_ corresponded to Pluronic in rabbit cornea. *P*_app_ values recorded for corneas were greater than those estimated for scleras. Among the species, the *P*_app_ values recorded in rabbits were greater than those obtained for bovine and porcine in both cornea and sclera. Except for sclera data recorded for Pluronic formulations, *P*_app_ values were higher when tested in porcine compared to bovine tissue (Table 5). Since there are no commercially available ocular formulations of PG, as a control a PG solution (343.04 μg/mL) was prepared from a commercially available PG: Methyl-β-cyclodextrin complex powder. Permeability tests carried out in rabbit cornea and sclera led to J values of 2.02 ± 0.92 and 4.86 ± 2.37 μg/(cm^2^·h), respectively. The permeability coefficient, *P*_app_, values were 16.2 ± 7.5 × 10^−7^ and 39.3 ± 19.1·× 10^−7^ cm/s for cornea and sclera, respectively. *P*_app_ values recorded in cornea were similar to those recorded for the polymeric micelle dispersions, but *P*_app_ values recorded in sclera were larger for the PG: Methyl-β-cyclodextrin complex. This finding may be related to the fact that the complex is smaller than a PG-loaded micelle, and thus it can cross the sclera porous network more easily [56].

Finally, the dependence of the permeability coefficient values on the thickness of cornea and sclera was analyzed (Figure S4 in Supplementary Materials). Except for the small PG-loaded Pluronic micelles that did not show a significant dependence of *P*_app_ on sclera thickness, all other formulations showed a negative correlation between *P*_app_ and the tissue thickness. In the absence of other mechanisms, drug penetration in ocular tissues may be driven by diffusion. Diffusion coefficient is well known to be inversely proportional to the thickness of the membrane and, although cornea and sclera have a non-homogenous structure and several mechanisms may interplay, the thickness of the tissue (particularly of the complex cornea) is an additional barrier to the pass of the drug. The obtained results are in good agreement with previous findings on that the lowest permeability coefficients corresponded to the thicker bovine cornea [26].

## 4. Conclusions

Soluplus and Pluronic F68 exhibited different abilities to encapsulate PG, which may be related to their different HLB. Pluronic micelles increased PG solubility one order of magnitude in a buffered pH 7.4 medium, while Soluplus formulations enhanced the apparent solubility by two orders of magnitude. Relevantly, the tested micelle dispersions, particularly those prepared with Soluplus 20% exhibited an encapsulation efficiency of approx. 100%, which indicates that PG has strong affinity for the micelle core. The wide range of PG that can be encapsulated in Pluronic and Soluplus micelles may offer an enormous versatility to formulate the dose required for each patient affected by retinitis pigmentosa. Importantly, all copolymer concentrations tested successfully passed the HET-CAM assay.

Relevant differences in PG permeability through cornea and sclera were observed depending on the copolymer used to prepare the formulations and the animal chosen as the source of the tissues. Regarding the copolymer, Pluronic micelles provided higher permeability coefficients (*P*_app_) which may be related to their few nanometers size and less stable micelle structure. Thus, depending on the clinical demands, Soluplus and Pluronic micelles may offer different performances; namely, Soluplus micelles can provide overall greater amounts of PG accumulated in cornea and sclera and transferred to the eye tissues, while Pluronic F68 promote a faster penetration of lower PG doses.

Regarding the interspecies differences, PG penetration through rabbit´s cornea occurred faster than through porcine or bovine cornea. In the case of sclera, although faster for rabbit, interspecies differences were more relevant for Soluplus micelles than for Pluronic micelles. This latter finding maybe related again to the greater size of Soluplus micelles and suggests that sclera tissues from different species may have a different pore network architecture in addition to different thickness. Moreover, the in situ gelling capability of Soluplus-based formulations may enhance the retention time on the eye surface. Overall, the developed micelle formulations can be pointed out as interesting tools for the ocular delivery of PG, and porcine tissues may provide a more balanced view of the performance of the formulations compared to the highly permeable rabbit tissues and the poorly permeable bovine tissues.

## Figures and Tables

**Table 1:** pH, particle size (number distribution), polydispersion index (PDI), and Z-potential (mV) of Soluplus and Pluronic (12%) micelles in a PBS pH 7.4 at 25 °C before and after being loaded with progesterone (PG). Mean values ± standard deviations; *n* = 3.

Formulation	pH	Particle Size (nm)	PDI	Z-Potential (mV)
Soluplus 12%	6.46	52.32 ± 10.13	0.24 ± 0.01	−1.17 ± 0.38
Soluplus 12% + PG	6.38	59.19 ± 0.41	0.24 ± 0.02	−1.67 ± 0.62
Pluronic F68 12%	7.39	3.70 ± 1.01	0.74 ± 0.08	−1.76 ± 1.50
Pluronic F68 12% + PG	7.30	3.52 ± 0.15	0.73 ± 0.07	−0.46 ± 0.50

**Table 2:** PG solubilization capacity of Soluplus dispersions in a PBS pH 7.4, calculated using Equations (1)–(6). SP: Soluplus; PG: Progesterone; χ: Molar solubilization capacity; log P: Logarithm of the partition coefficient; PM: Molar partition coefficient; ∆G: Gibbs free standard solubilization energy; mf: Molar fraction of the encapsulated drug within the micelle.

Copolymer (% *w*/*w*)	SP (M)	PG (M)	PG (µg/mL)	χ	log P	PM	∆G for PM (KJ/mol)	mf
4	3.47 × 10^−4^	2.74 × 10^−4^	86.25	7.89 × 10^−1^	4.29	56,163,941.1	−44,211.8	0.99995
8	6.95 × 10^−4^	4.74 × 10^−4^	149.12	6.82 × 10^−1^	4.53	48,546,060.2	−43,850.6	0.99997
12	1.04 × 10^−3^	6.88 × 10^−4^	216.50	6.60 × 10^−1^	4.69	46,987,265.0	−43,769.7	0.99998
16	1.39 × 10^−3^	8.13 × 10^−4^	255.77	5.85 × 10^−1^	4.76	41,631,784.1	−43,469.9	0.99998
20	1.73 × 10^−3^	8.21 × 10^−4^	258.18	4.72 × 10^−1^	4.77	33,619,107.1	−42,940.3	0.99998

**Table 3:** PG solubilization capacity of Pluronic F68 dispersions in a PBS pH 7.4, calculated using Equations (1)–(6). PL F68: Pluronic F68; PG: Progesterone; χ: Molar solubilization capacity; log P: Respect of the partition coefficient; PM: Molar partition coefficient; ∆G: Gibbs free standard solubilization energy; mf: Molar fraction of the encapsulated drug within the micelle.

Copolymer (% *w*/*w*)	PL F68 (M)	PG(M)	PG (µg/mL)	χ	log P	PM	∆G for PM (KJ/mol)	mf
4	4.79 × 10^−3^	3.07 × 10^−5^	9.67	6.47 × 10^−3^	3.34	460,565.1	−32,309.9	0.99954
8	9.58 × 10^−3^	3.52 × 10^−5^	11.08	3.69 × 10^−3^	3.40	262,942.2	−30,921.1	0.99960
12	1.44 × 10^−2^	5.47 × 10^−5^	17.21	3.82 × 10^−3^	3.59	271,837.0	−31,003.5	0.99974
16	1.92 × 10^−2^	9.60 × 10^−5^	30.18	5.02 × 10^−3^	3.83	357,388.1	−31,681.5	0.99985
20	2.39 × 10^−2^	1.49 × 10^−5^	46.81	6.23 × 10^−3^	4.02	443,288.4	−32,215.2	0.99991

**Table 4:** Accumulated amounts of PG in the receptor compartments at 240 min in diffusion tests from 20% copolymer micelle dispersion using rabbit, porcine, and bovine cornea and sclera.

Micelles	Accumulated Amounts of PG in Receptor Compartments (μg/cm^2^)					
	Cornea			Sclera		
	Rabbit	Porcine	Bovine	Rabbit	Porcine	Bovine
Soluplus	3.65 ± 0.50	2.44 ± 0.17	1.13 ± 0.09	2.30 ± 0.64	1.12 ± 0.36	0.83 ± 0.02
Pluronic	1.75 ± 0.48	0.77 ± 0.13	0.92 ± 0.06	0.71 ± 0.13	0.36 ± 0.23	0.82 ± 0.03

**Table 5:** Flux (*J*, μg/(cm^2^∙h)) and permeability coefficient (*P*_app_, cm/s) in rabbit, pig, and cow cornea and sclera for PG formulated in Soluplus and Pluronic micelles (*n* = 4). The total polymer concentration was 20% *w*/*w* in all cases (*n* = 4).

Animal	Soluplus/Cornea		Soluplus/Sclera		Pluronic/Cornea		Pluronic/Sclera	
	*J*	*P*_app_ (×10^7^)	*J*	*P*_app_ (×10^7^)	*J*	*P*_app_ (×10^7^)	*J*	*P*_app_ (×10^7^)
Rabbit	1.38 ± 0.15	16.5 ± 1.8	0.77 ± 0.17	9.2 ± 2.0	0.66 ± 0.19	37.3 ± 10.5	0.26 ± 0.03	14.5 ± 1.5
Pig	0.91 ± 0.04	9.8 ± 0.5	0.37 ± 0.08	4.0 ± 0.9	0.26 ± 0.04	15.2 ± 2.3	0.09 ± 0.07	5.6 ± 3.9
Cow	0.31 ± 0.07	3.4 ± 0.8	0.11 ± 0.01	1.2 ± 0.1	0.10 ± 0.03	6.1 ± 1.5	0.11 ± 0.01	6.8 ± 0.8

## Supplementary Materials

The following are available online at https://www.mdpi.com/1999-4923/12/8/702/s1, Table S1: Particle size of micelles of Soluplus and Pluronic micelles in phosphate saline buffer (PBS) pH 7.4 at 25 °C before and after being loaded with progesterone (PG). Mean values ± standard deviations; *n* = 3, Figure S1: DLS size distributions in number for selected micelle dispersions recorded at 25 °C. Figure S2: Transmittance at room temperature of 20% *w*/*w* Soluplus dispersion prepared in PBS pH 7.4 and measured using 10-mm light path cuvette, Figure S3: Appearance of the viscous 20% *w*/*w* Soluplus dispersion in PBS pH 7.4 at 37 °C, Figure S4: Dependence of the apparent permeation coefficient (*P*_app_) of PG on the thickness of cornea (orange and green lines) and sclera (pink and purple lines) when administered formulated in Pluronic F68 or Soluplus micelles. Thickness increased in the order rabbit < porcine < bovine tissue.
