# Supplementary material for: Micelles of Progesterone for Topical Eye Administration: Interspecies and Intertissues Differences in Ex Vivo Ocular Permeability
Source: Pharmaceutics. 2020 Jul 26;12(8):702. doi: 10.3390/pharmaceutics12080702 (PMC7464168; doi:10.3390/pharmaceutics12080702)
Supplement: Supplementary file 1 [file pharmaceutics-12-00702-s001.pdf]

Supplementary Materials: Micelles of Progesterone for Topical Eye Administration: Interspecies and Intertissues Differences in Ex Vivo Ocular Permeability

Adrián M. Alambiaga-Caravaca, María Aracely Calatayud-Pascual, Vicent Rodilla, Angel Concheiro, Alicia López-Castellano and Carmen Alvarez-Lorenzo *

Table S1. Particle size of micelles of Soluplus and Pluronic micelles in phosphate saline buffer (PBS) pH 7.4 at 25 °C before and after being loaded with progesterone (PG). Mean values ± standard deviations; *n* = 3.

Copolymer (% *w*/*w*)	SP	SP + PG	PL	PL + PG
4	51.23 ± 0.62	53.45 ± 2.68	5.49 ± 0.88	4.35 ± 0.17
8	53.33 ± 1.07	48.72 ± 0.48	2.94 ± 1.07	4.36 ± 0.56
12	52.32 ± 10.13	59.19 ± 0.41	3.70 ± 1.01	3.52 ± 0.15
16	60.89 ± 12.94	72.09 ± 3.67	3.22 ± 0.49	3.36 ± 0.02
20	53.58 ± 5.05	66.18 ± 21.32	3.32 ± 0.13	2.20 ± 0.15

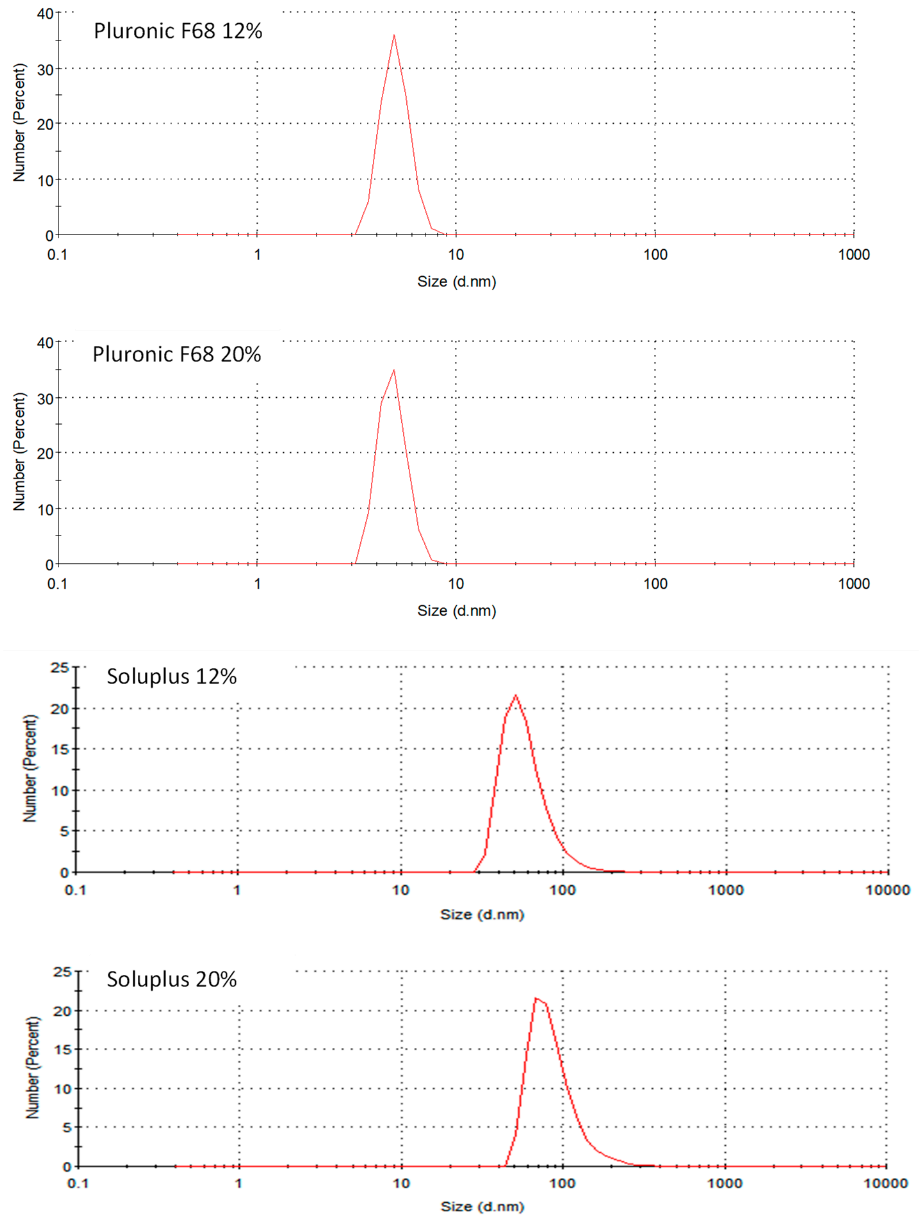

Figure S1. DLS size distributions in number for selected micelle dispersions recorded at 25 °C.

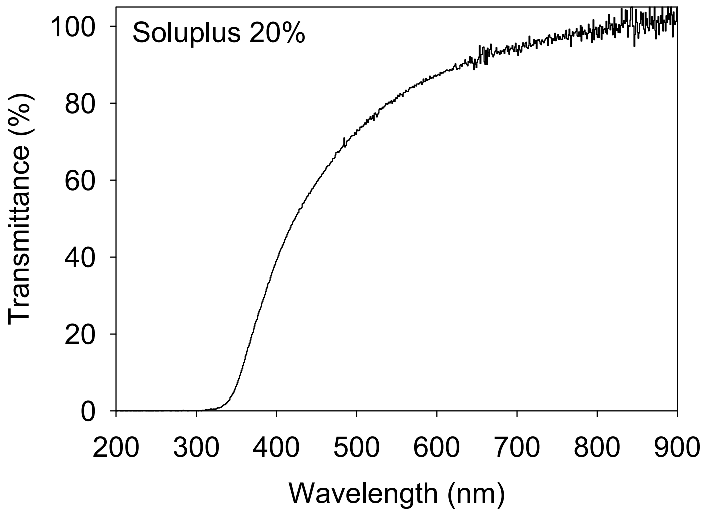

Figure S2. Transmittance at room temperature of 20% *w*/*w* Soluplus dispersion prepared in PBS pH 7.4 and measured using 10-mm light path cuvette.

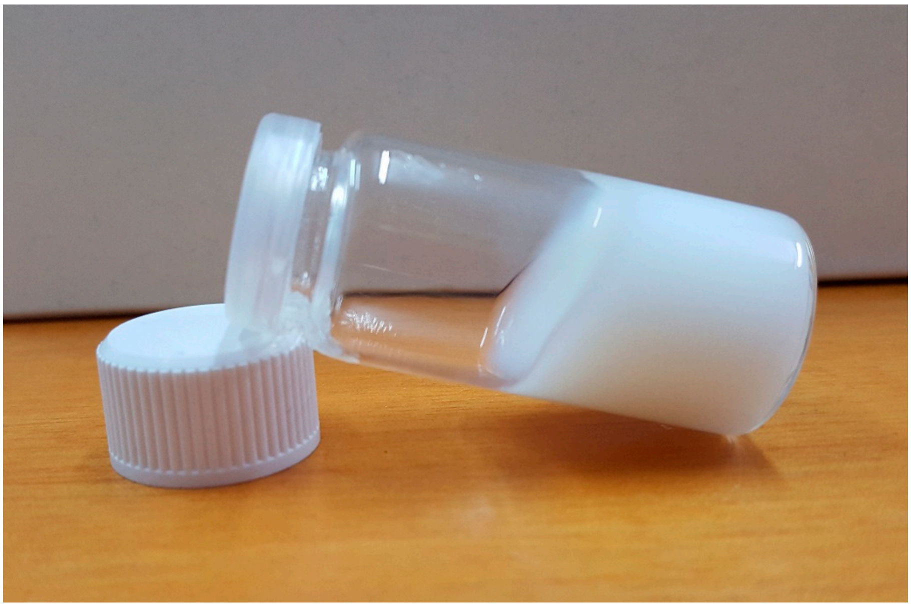

Figure S3. Appearance of the viscous 20% *w*/*w* Soluplus dispersion in PBS pH 7.4 at 37 °C.

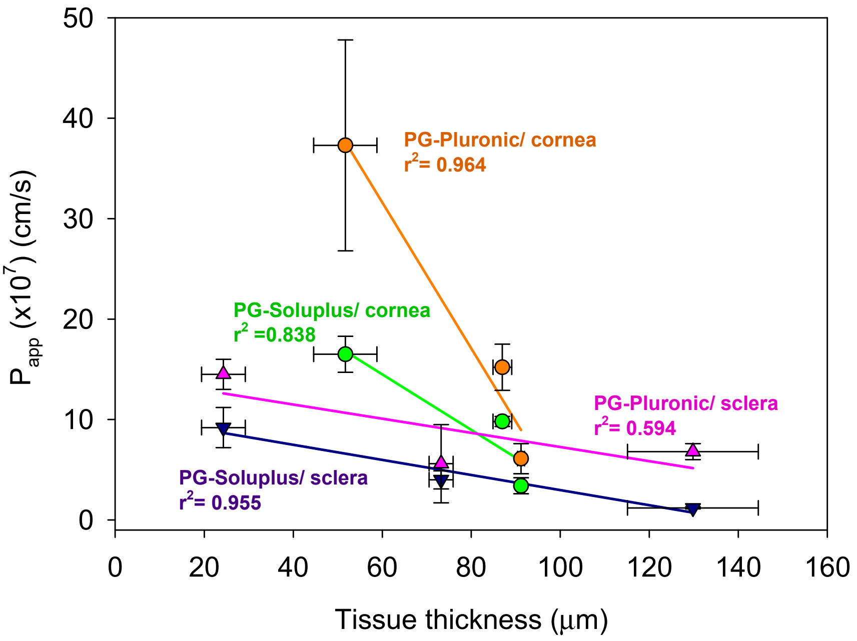

Figure S4. Dependence of the apparent permeation coefficient (P_{app}) of PG on the thickness of cornea (orange and green lines) and sclera (pink and purple lines) when administered formulated in Pluronic F68 or Soluplus micelles. Thickness increased in the order rabbit < porcine < bovine tissue.
